# Hyperfunction of CD4 CD25 regulatory T cells in de novo acute myeloid leukemia

**DOI:** 10.1186/s12885-020-06961-8

**Published:** 2020-05-26

**Authors:** Yuling Wan, Congxiao Zhang, Yingxi Xu, Min Wang, Qing Rao, Haiyan Xing, Zheng Tian, Kejing Tang, Yingchang Mi, Ying Wang, Jianxiang Wang

**Affiliations:** State Key Laboratory of Experimental Hematology, National Clinical Research Center for Blood Diseases, Institute of Hematology & Blood Diseases Hospital, Chinese Academy of Medical Sciences & Peking Union Medical College, No. 288, Nanjing Road, Tianjin, 300020 China

**Keywords:** Regulatory T cells, Regulatory B cells, Acute myeloid leukemia, Tumor immunity, Immune escape

## Abstract

**Background:**

Acute myeloid leukemia (AML) is a common hematopoietic malignancy that has a high relapse rate, and the number of regulatory T cells (Tregs) in AML patients is significantly increased. The aim of this study was to clarify the role of Tregs in the immune escape of acute myeloid leukemia.

**Methods:**

The frequencies of Tregs and the expression of PD-1, CXCR4 and CXCR7 were examined by flow cytometry. The expression of CTLA-4 and GITR was tested by MFI. Chemotaxis assays were performed to evaluate Treg migration. The concentrations of SDF-1α, IFN-γ and TNF-α were examined by ELISA. Coculture and crisscross coculture experiments were performed to examine Treg proliferation and apoptosis and the effect of regulatory B cells (Breg) conversion.

**Results:**

The frequencies of Tregs in peripheral blood and bone marrow in AML patients were increased compared with those in healthy participants. AML Tregs had robust migration towards bone marrow due to increased expression of CXCR4. AML Treg-mediated immunosuppression of T cells was achieved through proliferation inhibition, apoptosis promotion and suppression of IFN-γ production in CD4^+^CD25^−^ T cells. AML Bregs induced the conversion of CD4^+^CD25^−^T cells to Tregs.

**Conclusion:**

In AML patients, the Breg conversion effect and robust CXCR4-induced migration led to Treg enrichment in bone marrow. AML Tregs downregulated the function of CD4^+^CD25^−^ T cells, contributing to immune escape.

## Background

Regulatory T cells (Tregs), which were originally identified as CD4^+^CD25^+^ T cells, are critical for maintaining immunological self-tolerance in healthy individuals by actively suppressing self-reactive lymphocytes [1]. However, in tumor immunity, Tregs are considered pivotal regulators of immune escape, as they suppress the proliferation and function of immune cells through cell-to-cell contact and inhibitory cytokine production [2]. Previous studies demonstrated that Tregs are increased in many solid tumors [3–6], as well as hematopoietic malignancies [7–9]. In addition, an increased Treg frequency is related to poor prognosis [6, 10, 11].

Acute myeloid leukemia (AML) is a hematopoietic malignancy driven by a sequence of somatic mutations in multipotential primitive cells or progenitor cells. Currently, although most AML patients achieve complete remission (CR) after induction chemotherapy, approximately 60% of patients who receive subsequent consolidation chemotherapy still cannot achieve long survival. As immunotherapy is a promising new therapeutic option for hematologic malignancies [12], Tregs can be a novel target. However, little is known regarding the role of Tregs in the immune escape of AML and the possible upstream mechanisms.

As cell-to-cell contact plays an essential role in the immunosuppression of Tregs [13], the accumulation of Tregs in bone marrow (BM) is crucial for AML progression. A previous study indicated that stromal cell-derived factor (SDF)-1α/chemokine (C-X-C motif) receptor (CXCR) 4 signaling plays an important role in regulating Treg levels in BM and peripheral blood (PB) [14]. Blockade of the SDF-1α/CXCR4 axis alone, which is crucial for Treg migration, reduces ovarian tumor growth and peritoneal dissemination and selectively reduces intratumoral Tregs [15]. Therefore, we hypothesize that excessive chemotaxis of Tregs from PB to BM is the reason for Treg enrichment. In addition, regulatory B cells (Bregs) are another negative regulator that supports immunological tolerance by producing interleukin-10 (IL-10), IL-35, and transforming growth factor β (TGF-β), as well as influencing the expansion of T cells [16]. Olkhanud et al. indicated that tumor-evoked Bregs promoted breast cancer metastasis by converting CD4^+^ T cells into Tregs using a mouse 4 T1 breast cancer model [17]. Accordingly, Bregs may play a similar role in inducing Treg conversion in AML immune escape.

To provide an in-depth understanding of Tregs in AML, in the present study, we investigated the proliferation, cytokine production and migration capacity of Tregs in newly diagnosed untreated AML patients.

## Methods

### Cell samples

EDTA anticoagulated bone marrow aspirates and peripheral blood samples were collected from 45 (24 male and 21 female) de novo AML patients with a median age of 36 years (range, 18–68 years) and 29 healthy donors (17 male and 12 female; age range, 21–56 years) from the Institute of Hematology & Blood Diseases Hospital of the Chinese Academy of Medical Sciences from March 2015 to April 2017.

### Antibodies and other agents

Manufacturer names are included in supplementary materials.

### Cell isolation and flow cytometry

Mononuclear cells from BM and PB were isolated by density gradient centrifugation (Ficoll, TBDscience). EDTA was used as an anticoagulant.

For surface markers, cells were stained with monoclonal PD1-APC/Cy7, CTLA4-PE, GITR-PE, CXCR4-PE or CXCR7-PE antibodies simultaneously with CD4-FITC, CD25-Pacific Blue and CD127-APC antibodies.

For intracellular FOXP3 expression, cells were fixed and permeabilized with FOXP3 fixation/permeabilization solution (Ebioscience) after staining with CD4-FITC, CD25-Pacific Blue and CD127-APC and were then stained with FOXP3-PE antibody.

For intracellular cytokine expression, cells were measured after stimulation with phorbol myristate acetate (PMA, 0.05 μg/ml; Sigma-Aldrich) and ionomycin (1 μg/ml, Sigma-Aldrich) in the presence of brefeldin A (BFA 0.01 μg/ml, BD Biosciences) at 37 °C for 5 h. After staining with CD19-FITC, CD24-PE and CD38-APC antibodies, the cells were fixed and permeabilized, followed by intracellular staining with IL-10-PE/Cy7 and TGF-β-PerCP/Cy5.5 antibodies according to the manufacturer’s instructions.

The concentration of antibodies was determined as recommended by the manufacturer. Isotype controls were used to set correct gating for both extracellular and intracellular markers. Gating strategies are presented in the supplementary materials. Data acquisition was performed on a Canto II flow cytometer (BD Biosciences) and analyzed by FlowJo software (Version 7.6; TreeStar).

### Purification of lymphocyte subpopulations

Three T cell subpopulations, including CD4^+^CD25^−^ T cells, CD8^+^ T cells, and CD4^+^CD25^+^ Tregs, were purified using a commercial CD3-positive selection magnetic activated cell sorting (MACS) isolation kit (Miltenyi Biotec), a CD8-positive selection isolation kit (Miltenyi Biotec) and a CD4^+^CD25^+^ human Treg isolation kit (Miltenyi Biotec) according to the manufacturer’s instructions. To verify the phenotype of Tregs, some sorted CD4^+^CD25^+^ Tregs were stained with CD4-FITC, CD25-Pacific Blue and CD127-APC antibodies and analyzed on a Canto II flow cytometer (BD Biosciences). Flow cytometry results showed that the purity of CD4^+^CD25^+^CD127^low/−^ Tregs was > 95%. For CD19^+^CD24^+^ Breg isolation, BM mononuclear cells were stained with CD19-FITC and CD24-PE antibodies and sorted on an Aria III flow cytometer (BD Biosciences) according to the manufacturer’s instructions, and the purity of isolated Bregs was > 95%.

### Chemotaxis assay

Purified PB Tregs were subjected to a migratory assay with SDF-1 or BM fluid as chemotactic media. Purified PB Tregs (1 × 10^5^ cells/well) were induced to migrate towards either SDF-1 (100 ng/mL; R&D Systems) or BM fluid diluted in RPMI 1640 with 10% fetal bovine serum (FBS) in a 24-well plate containing 5-μm pore polycarbonate filters (Costar Corporation). After incubation at 37 °C for 3 h, migrating cells were harvested from the lower compartment. The harvested cells were enumerated using a hemocytometer. The percentage of migrating cells was calculated by determining the ratio of the number of cells harvested from the lower compartment to the total number of cells loaded in the upper compartment. CXCR4 neutralization experiments were performed by incubating the cells with AMD3100 (100 ng/mL; Sigma-Aldrich) for 30 min before adding the cells to the top chamber.

### Proliferation assays for T cells

To assess the proliferation of T cells, freshly purified CD4^+^CD25^−^ T cells or CD8^+^ T cells were stained with 5 mmol/L carboxyfluorescein succinimidyl ester (CFSE) (Invitrogen) according to the manufacturer’s instructions. The stained cells were then cultured in a 24-well plate alone or with Tregs at a ratio of 4:1 at 37 °C with 5% CO_2_ in X-VIVO™15 medium (Lonza) supplemented with 5% FBS and recombinant interleukin-2 (100 units/ml; R&D systems) and stimulated with anti-CD3/CD28 beads (Miltenyi Biotec) at a ratio of 1:1. After 5 days, proliferating CD4^+^CD25^−^ T cells or CD8^+^ T cells were identified as CFSE-diluted subsets. The control group consisted of CD4^+^CD25^−^ T cells that were not cocultured with Tregs but were stained with CFSE.

### Apoptosis assays for T cells

To assess the apoptosis of T cells, freshly purified CD4^+^CD25^−^ T cells or CD8^+^ T cells were cultured in a 24-well plate alone or with Tregs at a ratio of 4:1 at 37 °C with 5% CO_2_ in X-VIVO™15 medium (Lonza) supplemented with 5% FBS and recombinant Interleukin-2 (100 units/ml; R&D Systems) and were stimulated with anti-CD3/CD28 beads (Miltenyi Biotec). After 3 days, apoptotic CD4^+^CD25^−^ T cells or CD8^+^ T cells were assayed by an Annexin v/PI apoptosis kit (BD Biosciences) according to the manufacturer’s instructions.

### Detection of IFN-γ, TNF-α and SDF-1 levels

Culture supernatants obtained from the apoptosis assays were analyzed for IFN-γ and TNF-α by ELISA (Pepro Tech). SDF-1 was also detected in BM fluid and serum by ELISA (Pepro Tech). The detection range of all three kits was 0–10 ng/ml according to the manufacturer’s instructions.

### Conversion of CD4^+^CD25^−^T cells to CD4^+^CD25^+^Foxp3^+^ Tregs

To assess the efficiency of Breg-mediated conversion of Tregs, freshly purified CD4^+^CD25^−^ T cells were cultured in a 24-well plate alone or with Bregs at a ratio of 1:1 at 37 °C with 5% CO_2_ in X-VIVO™15 medium (Lonza) supplemented with 5% FBS and recombinant Interleukin-2 (100 units/ml; R&D Systems) and were stimulated with anti-CD3/CD28 beads (Miltenyi Biotec). After 5 days, the cells were analyzed for the expression of CD4, CD25 and Foxp3 on a Canto II flow cytometer (BD Biosciences).

### Statistical analysis

The data are shown as the mean ± SEM or median (P25, P75). The data were tested for normality, assuming the test result was *P* > 0.10. Statistical significance of differences between groups was determined by Student’s t tests. Nonnormally distributed data were analyzed by the Mann-Whitney U test. A value of *P* < 0.05 was determined to be statistically significant. Analyses were carried out with SPSS 16.0 software (SPSS Science).

## Results

### Increased frequencies of Tregs in AML patients

**Fig. 1:**
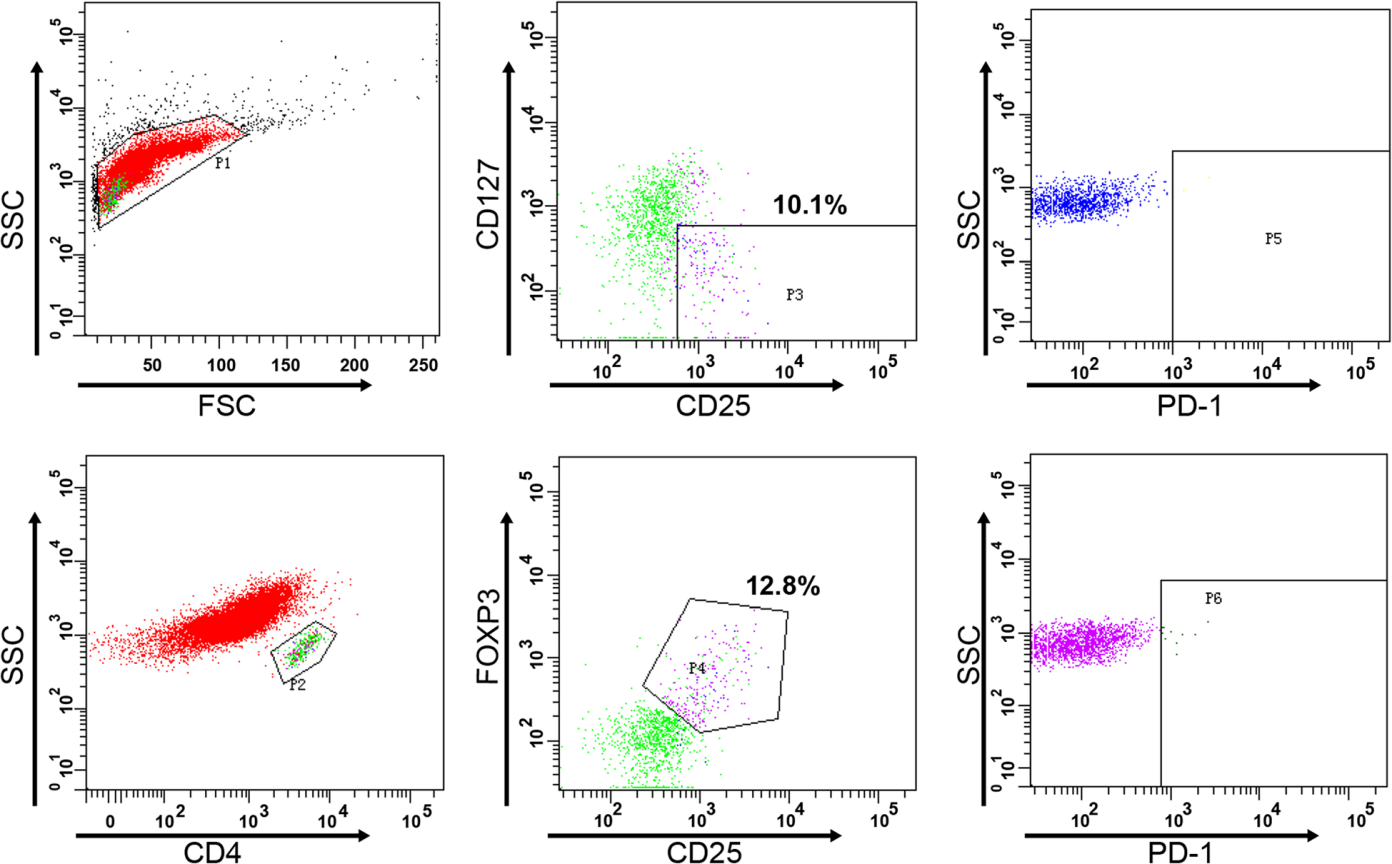
Flow cytometry analysis of CD4^+^CD25^+^CD127^low/-^ and CD4^+^CD25^+^Foxp3^+^ Tregs in BM from newly diagnosed AML patients

**Fig. 2:**
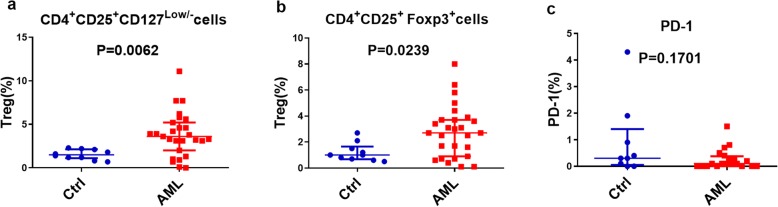
Increased frequencies of Tregs in AML patients. **a**. Frequencies of CD4^+^CD25^+^CD127^low/-^ Tregs in BM of AML patients (n=27) and healthy controls (n=10). **b**. Frequencies of CD4^+^CD25^+^Foxp3^+^ Tregs in BM of AML patients (n=27) and healthy controls (n=10). **c**. Frequencies of PD-1 on the surface of Tregs in AML patients (n=9) and healthy controls (n=20)

We used CD4^+^CD25^+^CD127^low/−^ as the immunophenotype of Tregs and verified this subgroup by FoxP3 expression. Flow cytometry analysis showed that CD4^+^CD25^+^CD127^low/−^ T cells and CD4^+^CD25^+^Foxp3^+^ T cells belonged to the same group (Fig. 1). Compared with those of healthy participants, the frequencies of CD4^+^CD25^+^CD127^low/−^ Tregs in the BM of AML patients were significantly increased (3.60% [range: 2.00 to 5.20%] vs 1.50% [range: 1.10 to 2.13%], *P* = 0.0062), as were CD4^+^CD25^+^Foxp3^+^ Tregs (2.70% [range: 0.90 to 3.70%] vs 1.00% [range: 0.68 to 1.65%], *P* = 0.0239) (Fig. 2a-b). However, the expression of programmed cell death 1 (PD-1) on the surface of Tregs in AML patients was not significantly different from that in healthy participants (*P* > 0.05) (Fig. 2c). Tregs with cytotoxic T lymphocyte-associated protein 4 (CTLA4) and glucocorticoid-induced tumor necrosis factor receptor (GITR) were undetectable in the analyzed samples.

### Enhanced migratory capacity of Tregs due to increased expression of CXCR4

**Fig. 3:**
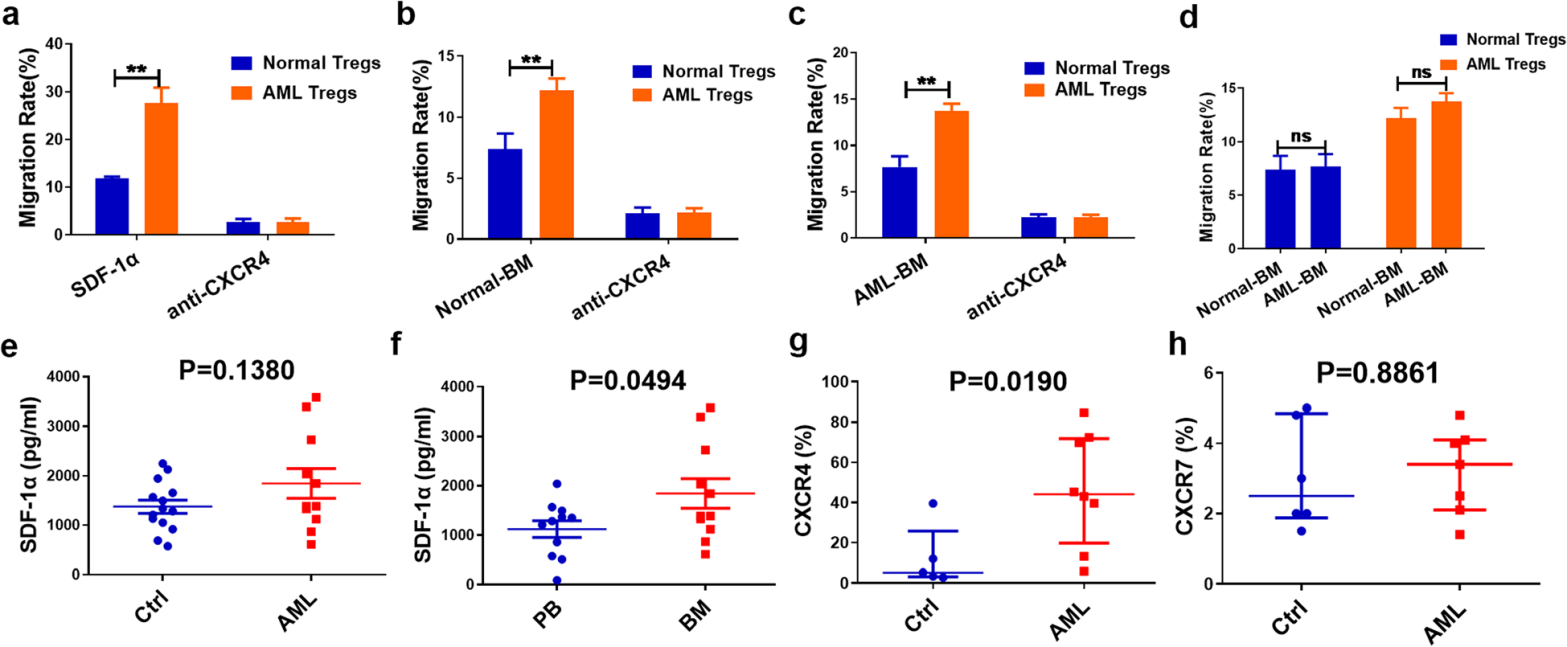
The enhancement of migratory capacity of Tregs due to higher expression of CXCR4 in AML. **a-c**. PB Tregs from AML patients (n = 3) had higher migratory capacity towards (a) SDF-1α, (b) normal BM, and (c) AML BM than those in controls (n = 3). CXCR4 blockade resulted in significantly reduced migratory capacity of Tregs in controls and AML, with no significant differences. **d**. The same PB Tregs showed similar migratory capacities to normal BM fluid or AML BM fluid. **e-f**. The level of SDF-1α in BM fluid from patients (n =1 1) and controls (n = 14) was similar (*P* = 0.1380). BM fluid had increased level of SDF-1 compared with serum in AML patients (n = 11). Data were expressed as mean ± SEM. ns *P* > 0.05, **P < 0.01. **g-h**. The expression of CXCR4 and CXCR7 on the surface of Tregs. There was significantly higher expression of CXCR4 on PB Tregs from AML patients (n = 8) compared with controls (n = 5) (*P* = 0.0310), while there was no significant difference in CXCR7 expression on PB Tregs between the 2 groups (AML patients n = 7; controls n = 6). Data were expressed as Median (P25, P75)

The ability of Tregs to suppress immune cells through contact-dependent mechanisms depends on their migratory capacity to the primary target tissue [18]. Therefore, excessive migration of Tregs to the BM could be the reason for Treg enrichment in the BM of AML patients. As SDF-1α/CXCR4 is crucial for Treg migration to BM and an alternate SDF-1α receptor, CXCR7, still needs to be investigated [14, 19], we first designed a chemotaxis assay to exclude the effect of other soluble chemoattractants in BM fluid from AML patients on Treg migration. SDF-1α, BM fluid from healthy participants and BM fluid from AML patients were used individually as chemotactic media. The results showed that PB Tregs from AML patients had stronger migration to all three media than normal Tregs (SDF-1α: 27.69 ± 1.84% vs. 11.73 ± 0.27%, *P* = 0.0010; normal BM: 12.20 ± 0.55% vs. 7.37 ± 0.75%, *P* = 0.0007; AML BM: 13.73 ± 0.47% vs. 7.67 ± 0.67%, *P* = 0.0018, respectively). Moreover, the same PB Tregs showed similar migratory capacities to normal BM fluid and AML BM fluid. After adding the CXCR4 blocker, the migration of PB Tregs from AML patients and controls was significantly decreased, with no significant differences (*P* > 0.05) (Fig. 3a-d).

The levels of SDF-1α in BM fluid from patients and controls were similar (1844 ± 300.2 ng/mL vs. 1374 ± 134.8 ng/mL, *P* = 0.1380) (Fig. 3e). In addition, BM fluid from AML patients had increased levels of SDF-1 compared with that of serum (1844 ± 300.2 ng/mL vs. 1123 ± 168.9 ng/mL, *P* = 0.0494) (Fig. 3f). To further investigate the factors influencing the migration of PB Tregs, flow cytometry was performed to detect the expression of CXCR4 and CXCR7 on Tregs. The results showed that the expression of CXCR4 on AML Tregs was significantly higher than that of the controls (44.15% [range: 19.78 to 71.78%] vs 5.20% [range: 3.10 to 25.75%], *P* = 0.0190), while the expression of CXCR7 showed no significant differences between the two groups (3.40% [range: 2.10 to 4.10%] vs 2.50% [range; 1.88 to 4.85%], *P* = 0.8861) (Fig. 3g-h).

### AML Treg-mediated immunosuppression of immune T cells

**Fig. 4:**
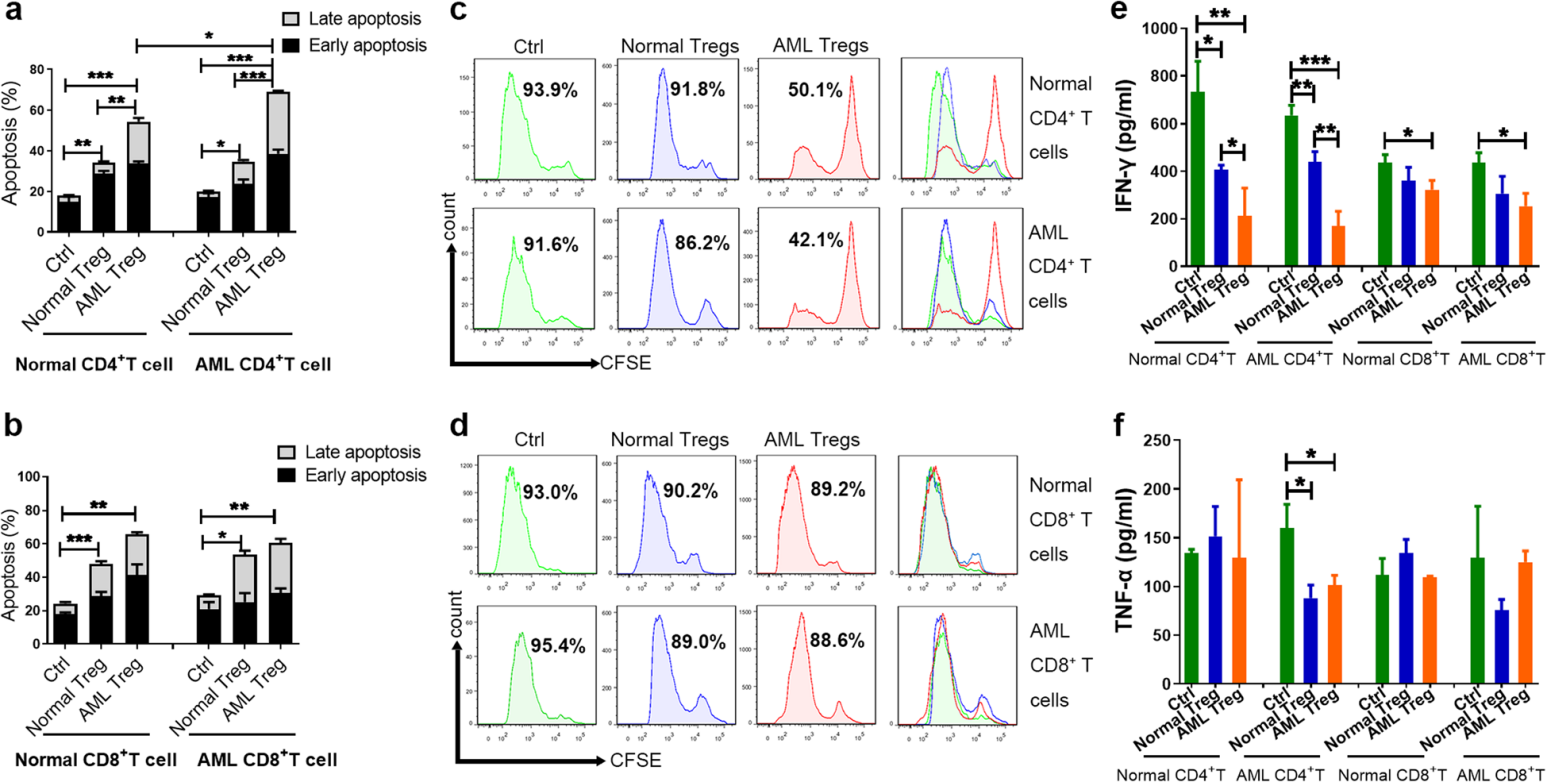
AML Treg-mediated immunosuppression for immune T cells. **a**. Both normal Tregs (n = 3) and AML Tregs (n = 3) resulted in increased apoptosis of T cells compared with CD4^+^CD25^-^ T cells cultured alone, particularly AML Tregs. **b**. Compared with normal Tregs (n = 3), AML Tregs (n = 3) had the same trend of progression of CD8^+^ T cells, however the differences remained not statistically significant (*P* > 0.05). **c**. AML Tregs inhibited the proliferation of CD4^+^CD25^-^ T cells (AML patients n = 3; controls n = 3). **d**. AML Tregs could not inhibit the proliferation of CD8^+^ T cells (AML patients n = 3; controls n = 3). **e**. The levels of IFN-γ showed that T cells from both normal controls and AML patients cocultured with AML Tregs had significantly lower levels of IFN-γ production than those of controls. CD8^+^ T cells showed the same trend, but the differences were not statistically significant. (AML patients n = 7; controls n = 6). **f**. The inhibitory effect was not observed for TNF-α (*P* > 0.05). (AML patients n = 7; controls n = 6). Data were expressed as mean ± SEM. *P < 0.05, **P < 0.01, ***P < 0.001

To study the immunosuppressive effects of Tregs on T cells, coculture and crisscross coculture experiments of Tregs with CD4^+^CD25^−^ T cells were performed. The results showed that Tregs from controls or AML patients promoted the apoptosis of normal CD4^+^CD25^−^ T cells compared with that of CD4^+^CD25^−^ T cells cultured alone (17.03 ± 1.97% vs. 34.40 ± 2.10%, *P* = 0.0038; 17.03 ± 1.97% vs. 55.27 ± 3.47%, *P* = 0.007). A similar trend was also found in AML CD4^+^CD25^−^ T cells (20.40 ± 1.69% vs. 32.47 ± 2.83%, *P* = 0.0215; 20.40 ± 1.69% vs. 68.93 ± 2.10%, *P* < 0.0001, respectively). Compared with normal Tregs, AML Tregs significantly promoted the apoptosis of both normal and AML CD4^+^CD25^−^ T cells (34.40 ± 2.10% vs. 55.27 ± 3.47%, *P* = 0.0068; 32.47 ± 2.83% vs. 68.93 ± 2.10%, *P* = 0.0005, respectively). In particular, AML Tregs had the most significant proapoptotic effect on AML CD4^+^CD25^−^ T cells (Fig. 4a). Tregs also promoted the apoptosis of CD8^+^ T cells (*P* < 0.05). There were no significant differences when separately comparing the different stages of apoptosis (Supplemental Figure 4A-D). However, the proapoptotic effect on CD8^+^ T cells seemed less different between normal and AML Tregs (*P* > 0.05) (Fig. 4b).

In addition, Tregs from AML patients inhibited the proliferation of both normal CD4^+^CD25^−^ T cells (proliferation of CD4^+^CD25^−^ T cells decreased from 91.8 to 50.1%) and AML CD4^+^CD25^−^ T cells (proliferation of CD4^+^CD25^−^ T cells decreased from 86.2 to 42.1%) compared with that of Tregs in the controls (Fig. 4c). However, no proliferation inhibition was observed for CD8^+^ T cells (proliferation of CD8^+^ T cells was approximately 90%) (Fig. 4d).

Interferon-γ (IFN-γ) is an important Th1-type cytokine [20]. Coculture supernatants obtained from the apoptosis assays were analyzed for IFN-γ, and the data revealed that Tregs from AML patients suppressed the secretion of IFN-γ by both normal and AML CD4^+^CD25^−^ T cells (*P* = 0.0065 and *P* = 0.0004, respectively), especially for autologous CD4^+^CD25^−^ T cells (the level of IFN-γ decreased from baseline value of 635.0 ± 24.7 pg/ml to 170.5 ± 35.3 pg/ml) (Fig. 4e). The same trend was observed for CD8^+^ T cells, but the differences were not statistically significant. In contrast, an inhibitory effect was not observed for the cytokine tumor necrosis factor α (TNF-α) (*P* > 0.05) (Fig. 4f).

### AML Breg-induced conversion of CD4^+^CD25^−^ T cells to CD4^+^CD25^+^Foxp3^+^ Tregs

**Fig. 5:**
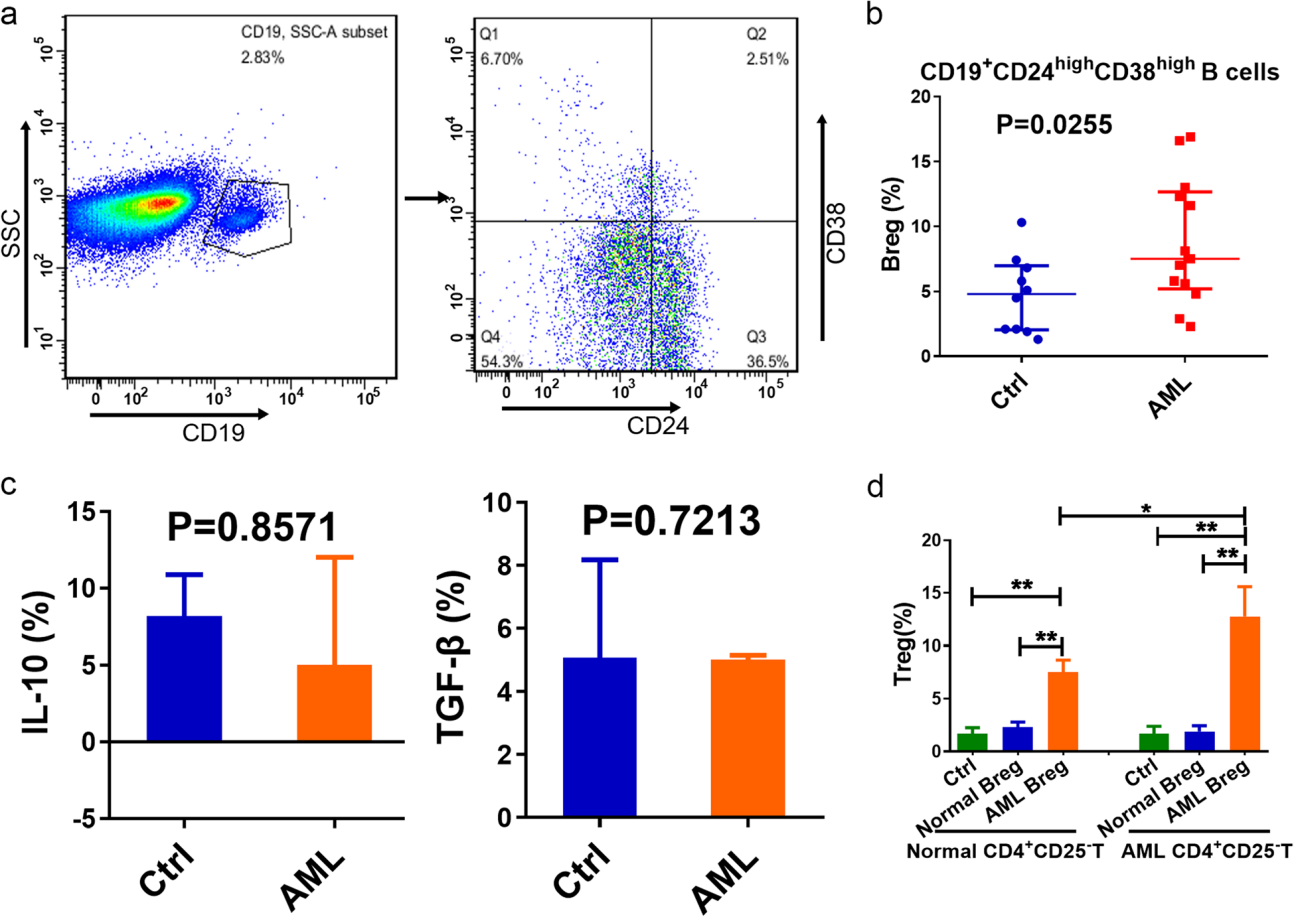
AML Breg-induced conversion of CD4^+^CD25^-^ T cells to CD4^+^CD25^+^Foxp3^+^ Tregs. **a**. Flow cytometry analysis of CD19^+^CD24^high^CD38^high^ Bregs in BM from newly diagnosed AML patients (n = 13). **b**. The percentage of CD19^+^CD24^high^CD38^high^ Bregs in BM from AML patients (n = 13) was increased compared with those from healthy controls (n = 10). **c**. The expression of IL-10 and TGF-β in BM Bregs cells had no significant differences between AML patients (n = 3) and controls (n = 4). Data were expressed as Median (P25, P75). **d**. Normal Bregs could not convert CD4+CD25- T cells into CD4^+^CD25^+^Foxp3^+^ Tregs whether from AML patients (n = 3) or controls (n = 4). AML Bregs could result in higher conversion of CD4^+^CD25^-^ T cells to CD4^+^CD25^+^Foxp3^+^ Tregs, especially for autologous CD4^+^CD25^-^ T cells. Data were expressed as mean ± SEM. *P < 0.05, **P < 0.01

Previous studies confirmed that in breast cancer, tumor-induced Bregs promote tumor metastasis by converting dormant CD4^+^CD25^−^ T cells into CD4^+^CD25^+^Foxp3^+^ Tregs, while in the absence of tumor-induced Bregs, the conversion of Tregs was significantly reduced and tumor metastasis was blocked [17]. To further address the role of Bregs in the conversion of Tregs, we first detected the frequencies of CD19^+^CD24^high^CD38^high^ Bregs in BM from newly diagnosed AML patients by flow cytometry (Fig. 5a). The percentage of CD19^+^CD24^high^CD38^high^ Bregs in BM CD19^+^ B cells from AML patients was significantly increased compared with those from healthy participants (7.50% [range: 5.20 to 12.65%] vs 4.80% [range: 2.05 to 6.95%], *P* = 0.0255) (Fig. 5b). We also detected intracellular cytokine expression, such as IL-10 and TGF-β, in Bregs from AML patients or healthy controls and found no significant differences between the two groups (*P* > 0.05) (Fig. 5c). Therefore, we considered whether this conversion was achieved through cell-to-cell contact by Bregs in AML patients. We cocultured Bregs with CD4^+^CD25^−^ T cells for 5 days in vitro, analyzed the expression of CD4, CD25 and Foxp3 by flow cytometry and found that normal Bregs cannot convert CD4^+^CD25^−^ T cells into CD4^+^CD25^+^Foxp3^+^ Tregs. Interestingly, the conversion of CD4^+^CD25^−^ T cells to CD4^+^CD25^+^Foxp3^+^ Tregs was significantly increased when normal or AML CD4^+^CD25^−^ T cells were cocultured with AML Bregs (2.31 ± 0.27% vs. 7.53 ± 0.65%, *P* = 0.0018; 1.89 ± 0.32% vs. 12.77 ± 1.63%, *P* = 0.0028). The conversion effect was most significant for autologous CD4^+^CD25^−^ T cells (Treg ratio increased from baseline value of 1.67 ± 0.34% to 12.77 ± 1.63%) (Fig. 5d).

## Discussion

AML accounts for more than 25% of adult leukemia cases [21]. In addition to the well-known cytogenetic and molecular genetic abnormalities in the pathogenesis of AML, immune escape also plays a significant role. Tregs are considered pivotal regulators of immune escape. As Tregs suppress the proliferation and function of immune cells through cell-to-cell contact [13], the enrichment of Tregs in tumor sites is crucial. In addition, using CD25-specific monoclonal antibodies to deplete Tregs in a variety of different mouse strains promoted the rejection of murine tumor cell lines, including melanoma and leukemia [22–24]. Previous studies have shown that compared to the frequencies in healthy individuals, the frequencies of Tregs in both PB and BM of AML patients are increased [25, 26]. Nevertheless, little is known about the mechanism of Tregs in the immune escape of AML.

FoxP3 is critical and specific for Treg identification. However, as FoxP3 is expressed intracellularly, and cells to be fixed and ruptured during staining, FoxP3 cannot be used to separate human Tregs for functional studies or in vivo expansion. In addition, CD127 is downregulated in Tregs and is negatively correlated with Foxp3 expression [27]. Therefore, we used CD4^+^CD25^+^CD127^low/−^ cells as the immunophenotype of Tregs and found that the frequencies of CD4^+^CD25^+^CD127^low/−^ Tregs in BM and PB were increased in AML patients. We deduced that excessive chemotaxis of Tregs from PB to BM is the reason for this enrichment. The homing of Tregs from PB to BM, which is the target tissue in AML, is crucial for their immunosuppressive function via direct contact with immune cells. The SDF-1α/CXCR4 signaling pathway is critical for Treg trafficking from PB to BM [14]. Moreover, the mechanism of CXCR7, another receptor of SDF-1α, still needs further investigation [19]. Our study demonstrated that the enrichment of Tregs in AML patients was due to excessive migration caused by increased expression of CXCR4 rather than abnormal expression of CXCR7 on Tregs or abnormal secretion of SDF-1α by the hematopoietic microenvironment.

Checkpoint inhibition was taken into consideration as contributing to the immunosuppressive effect of Tregs in AML. A series of inhibitory membrane proteins on the surface of Tregs can exert their immunosuppressive function through cell-to-cell contact [28]. However, in our experiments, differences in the expression of PD-1 on the surface of Tregs [10, 29], were not significantly different between AML and healthy participants, which was consistent with several solid cancers [30–32]. Therefore, we inferred that the immunosuppressive function of Tregs in AML patients does not rely on the overexpression of PD-1.

In addition, to further investigate the effects of Tregs on other immune cells, we designed in vitro coculture and crisscross coculture experiments. Our study revealed that compared to normal Tregs, AML Tregs were more capable of inhibiting proliferation, promoting apoptosis and suppressing the production of IFN-γ in both normal and AML CD4^+^CD25^−^ T cells. In particular, Tregs from AML patients had the most significant effect on autologous CD4^+^CD25^−^ T cells. Interestingly, the inhibitory effect on CD8^+^ T cells was not significantly different from that of controls. Although IFN- γ production by CD8^+^ T cells showed a similar decreasing trend, the difference was not obvious enough (*P* > 0.05). In ovarian cancer, a decreased ratio of CD8^+^ T cells to Tregs in tumors is related to poor prognosis, indicating suppression of effector CD8^+^ T cells by Tregs [1]. The effect of Tregs on CD8^+^ T cells in AML could be due to other mechanisms than cell-to-cell contact. Inhibitory cytokines such as TGF-β suppress CD8^+^ T cell proliferation [33], and cytokines could be the manner by which Tregs influence CD8^+^ T cells. In addition, as the function of CD8^+^ T cells relies on the help from CD4^+^ T cells, the immunosuppressive effect could be indirect via CD4^+^CD25^−^ T cells. Crisscross coculture experiments further ruled out the possibility that T cells had decreased resistance to Tregs. Therefore, we suggest that in AML, Treg-mediated immunosuppression of CD4^+^CD25^−^ T cells increases, leading to reduced proliferation, increased apoptosis and impaired secretion of IFN-γ; instead, the resistance of immune T cells to Tregs decreases, which eventually causes immune escape of AML cells.

The in-depth study of AML Tregs raised the question of the possible upstream factors in the initiation of AML Treg abnormalities. Mizoguchi et al. [34]. found a B cell subgroup that secreted IL-10 and inhibited the progression of inflammatory bowel disease, formally suggesting the concept of Bregs. Bregs are involved in the development of various diseases, including autoimmune diseases, infections and tumors. These cells are involved in the regulation of graft-versus-host disease (GVHD), mainly through the secretion of IL-10, TGF-β and other cytokines [35], regulation of T cells, amplification of Tregs [36–38] and other means of participating in immune regulation. A study showed that the number of Bregs in the PB of liver cancer patients was higher than that in healthy individuals [39]. Olkhanud et al. discovered that Bregs could convert dormant CD4^+^CD25^−^ T cells into CD4^+^CD25^+^Foxp3^+^ Tregs in breast cancer [17]. In our study, the percentage of CD19^+^CD24^high^CD38^high^ Bregs in BM was significantly increased. A coculture experiment proved that AML-induced Bregs robustly converted CD4^+^CD25^−^ T cells to CD4^+^CD25^+^Foxp3^+^ Tregs, while normal Bregs did not. However, as secretion of IL-10 and TGF-β did not show obvious differences between AML and normal Bregs, we deduced that IL-10 and TGF-β were not the reason for the conversion. Little research has been conducted on the impact of Bregs on Tregs. In breast cancer, PD-1^+^ Bregs increase the conversion [40]. Although that study did not use PD-1^−^ Bregs or anti-PD-1 antibodies for comparison, checkpoint inhibitors are still a good entry point for Breg investigations. The conversion effect was most significant when AML Bregs were cocultured with AML CD4^+^CD25^−^ T cells. Therefore, tumor-induced CD4^+^CD25^−^ T cells were more prone to conversion.

## Conclusion

In AML bone marrow, the frequencies of Bregs increase and induce the conversion of CD4^+^CD25^−^T cells to CD4^+^CD25^+^Foxp3^+^ Tregs. On the other hand, more PB Tregs home to the BM, which also causes the enrichment of Tregs in the BM. Treg-mediated immunosuppression in immune cells increases, leading to reduced proliferation and increased apoptosis and secretion of IFN-γ, especially for CD4^+^CD25^−^T cells.

## Supplementary information

**Additional file 1:** Manufacturer names of agents.

**Additional file 2:** Supplemental figures.

**Additional file 3:** Diagnostic information of participants.

## Data Availability

All data generated or analyzed during the present study are included in this published article. The authors declare that materials described in the manuscript, including all relevant raw data, will be freely available to any scientist wishing to use them for non-commercial purposes, without breaching participant confidentiality.

## Abbreviations

AML: Acute myeloid leukemia
Tregs: Regulatory T cells
Bregs: Regulatory B cells
CR: Complete remission
BM: Bone marrow
SDF-1α: Stromal cell-derived factor 1α
CXCR4: Chemokine (C-X-C motif) receptor 4
PB: Peripheral blood
IL-10: Interleukin-10
TGF-β: Transforming growth factor β
PMA: Phorbol myristate acetate
BFA: Brefeldin A
MACS: Magnetic activated cell sorting
FBS: Fetal bovine serum
CFSE: Carboxyfluorescein succinimidyl ester
PD-1: Programmed cell death protein-1
CTLA4: Cytotoxic T-lymphocyte-associated protein 4
GITR: Glucocorticoid-induced TNFR family–related protein
IFN-γ: Interferon-γ
TNF-α: Tumor necrosis factor α
GVHD: Graft-versus-host disease
HLA: Human leukocyte antigen
TCR: T cell receptor
