# Supplementary material for: Hyperfunction of CD4 CD25 regulatory T cells in de novo acute myeloid leukemia
Source: BMC Cancer. 2020 May 26;20:472. doi: 10.1186/s12885-020-06961-8 (PMC7249438; doi:10.1186/s12885-020-06961-8)
Supplement: Supplementary file 2 — Additional file 2: Supplemental figures. [file 12885_2020_6961_MOESM2_ESM.docx]

**Supplemental Figures**


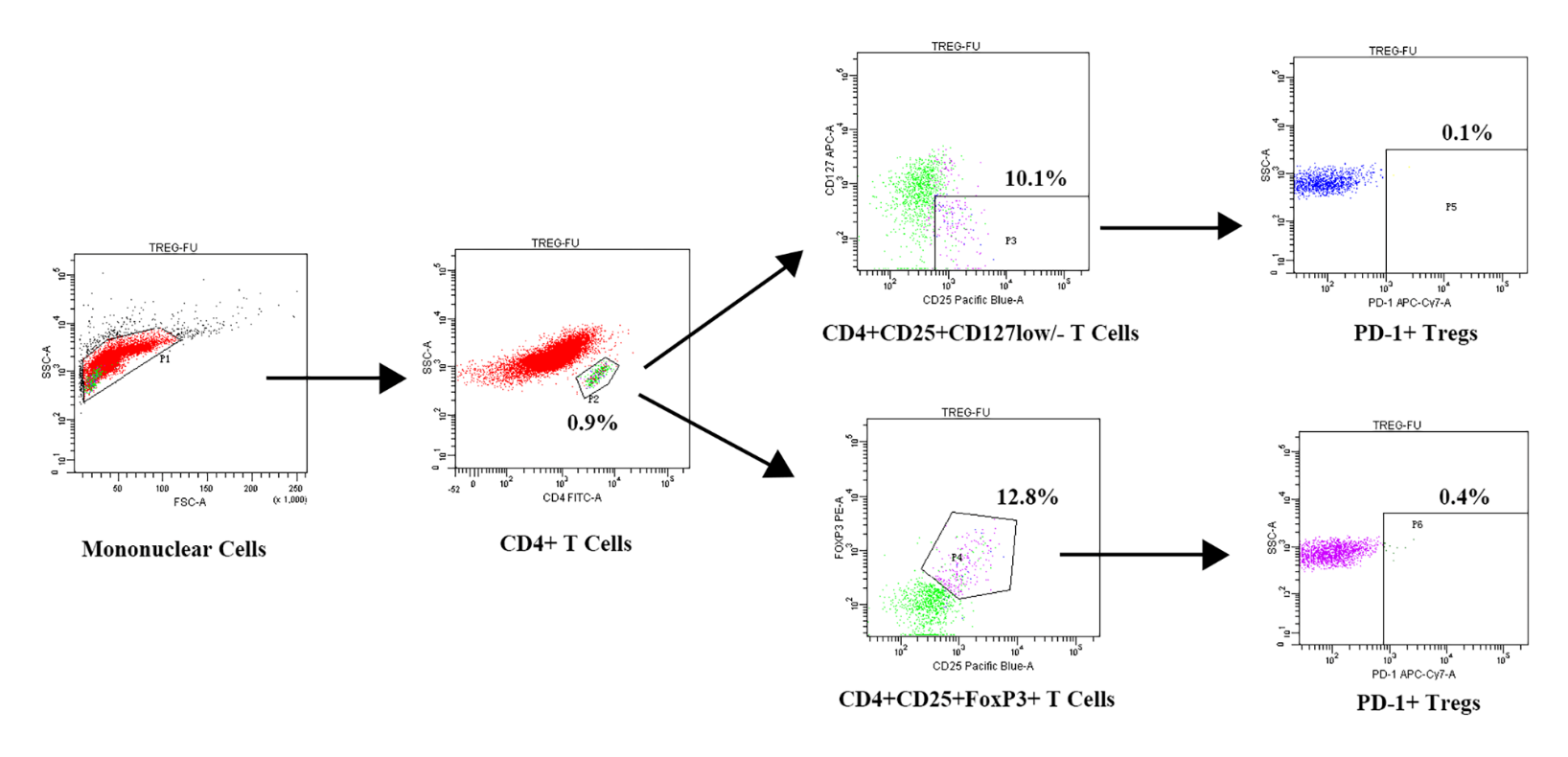


**Supplemental Figure 1. Gating strategies for Tregs and PD-1^+^ Tregs.**

Mononuclear cells obtained from peripheral blood and bone marrow of participants were gated on the CD4^+^ population. Gating for CD25^+^CD127^low/-^ and CD25^+^FoxP3^+^ cells indicated that the two phenotypes belonged to the same group. Isotype controls were used to set correct gating for both extracellular and intracellular markers.

**
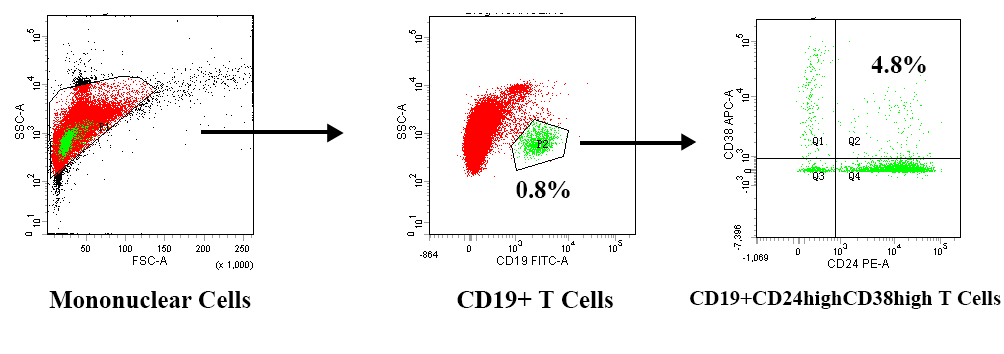
**

**Supplemental Figure 2. Gating strategies for Bregs.**

Mononuclear cells obtained from peripheral blood and bone marrow of participants were gated on the CD8^+^ population. Then separated the CD24^high^CD38^high^ subgroup.


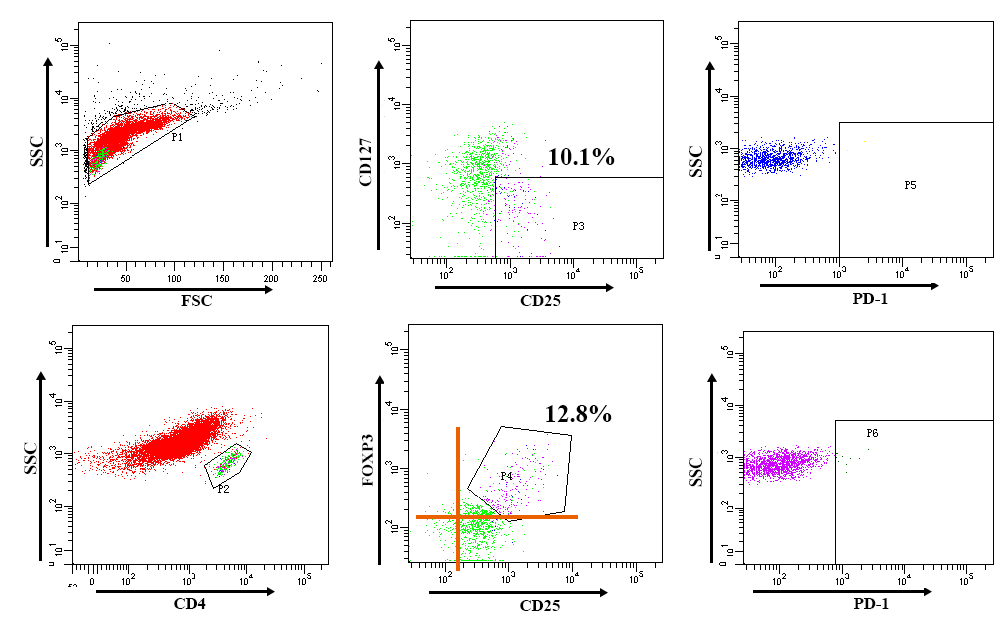


**Supplemental Figure 3. The frequency of FOXP3^+^ cells in CD4^+^CD25^-^ fraction**

As showed in Figure 1, in the second quadrant divided by orange line, the frequency of FOXP3^+^ cells in CD4^+^CD25^-^ T cells was quite low.


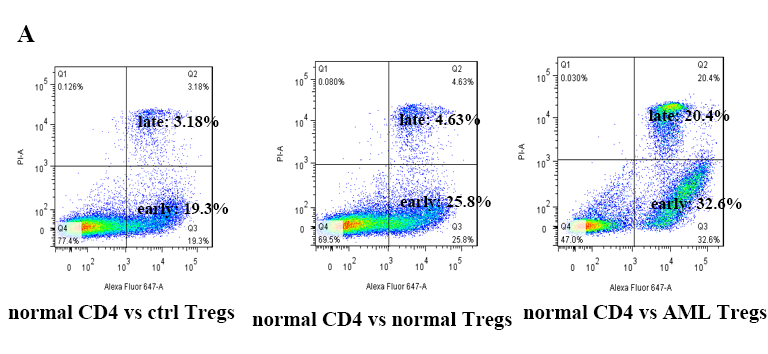


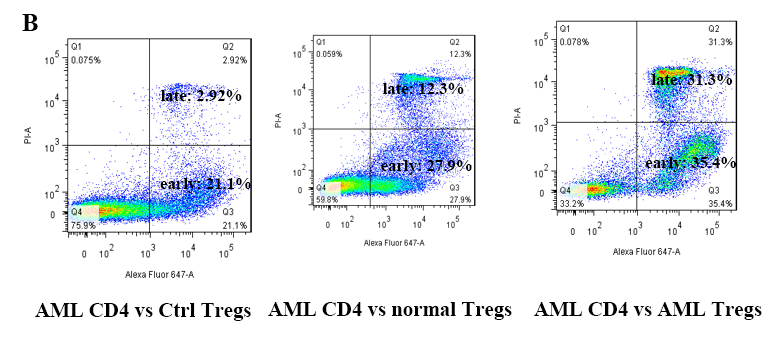


**
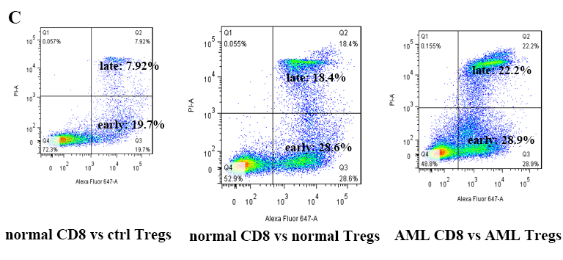
**

**
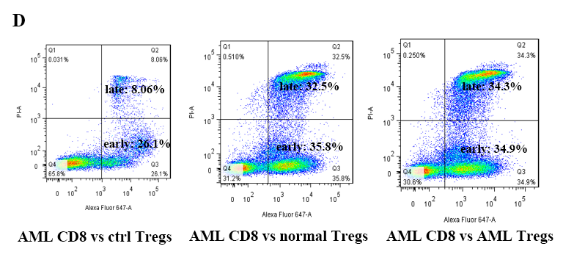
**

**Supplemental Figure 4. Frequencies of apoptotic cells in** **apoptosis assay.**

A-B. Apoptosis effect when CD4^+^CD25^-^ T cells cultured with Tregs. C-D. Apoptosis effect when CD8^+^ T cells cultured with Tregs. Frequencies of CD4^+^CD25^-^ and CD8^+^ T cells were marked in the figures. Early stage of apoptosis identified as forth quadrant, while late stage of apoptosis identified as first quadrant.

**
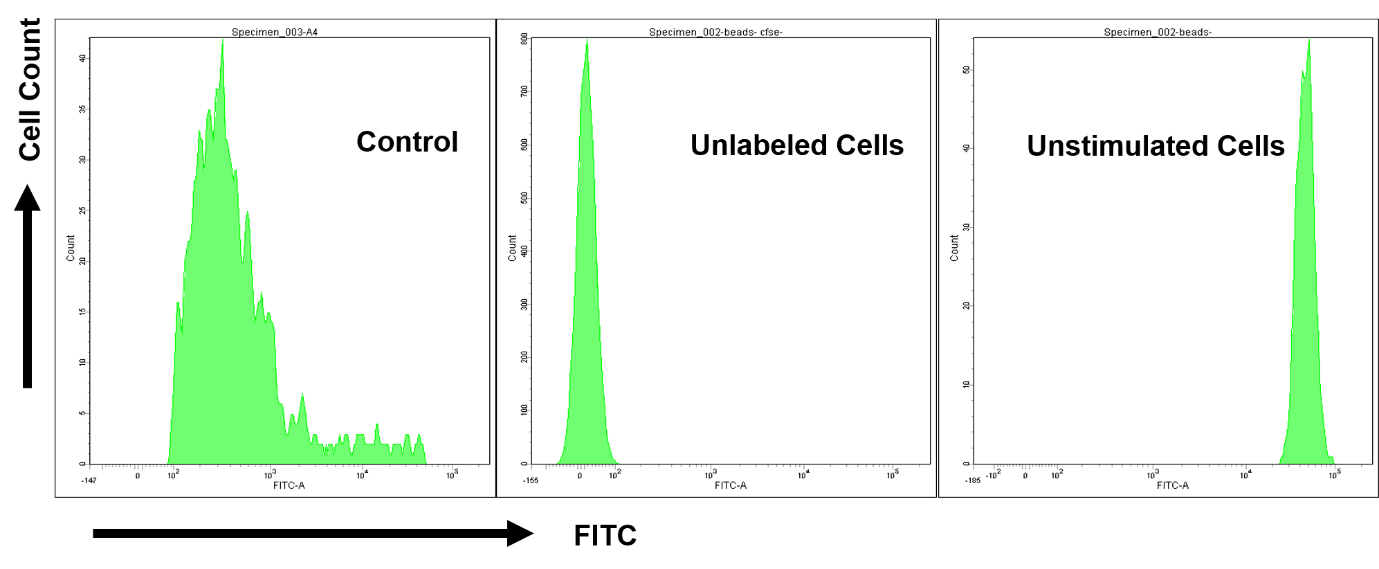
**

**Supplemental Figure 5. Gating strategies for proliferation analysis.**

The control was set as CD4^+^CD25^-^ T cells which did not be cocultured with Tregs but stained with CFSE, as the baseline control for proliferation. Control group showed stronger fluorescence intensity decay than test groups. Unstimulated cells were cells which did not be stimulated by with anti-CD3/CD28 beads, showed strongest fluorescence intensity.
